# Fractured Genetic Connectivity Threatens a Southern California Puma (*Puma concolor*) Population

**DOI:** 10.1371/journal.pone.0107985

**Published:** 2014-10-08

**Authors:** Holly B. Ernest, T. Winston Vickers, Scott A. Morrison, Michael R. Buchalski, Walter M. Boyce

**Affiliations:** 1 Wildlife Health Center, School of Veterinary Medicine, University of California Davis, Davis, California, United States of America; 2 Wildlife and Ecology Unit, Veterinary Genetics Laboratory, School of Veterinary Medicine, University of California Davis, Davis, California, United States of America; 3 The Nature Conservancy, San Francisco, California, United States of America; Macquarie University, Australia

## Abstract

Pumas (*Puma concolor;* also known as mountain lions and cougars) in southern California live among a burgeoning human population of roughly 20 million people. Yet little is known of the consequences of attendant habitat loss and fragmentation, and human-caused puma mortality to puma population viability and genetic diversity. We examined genetic status of pumas in coastal mountains within the Peninsular Ranges south of Los Angeles, in San Diego, Riverside, and Orange counties. The Santa Ana Mountains are bounded by urbanization to the west, north, and east, and are separated from the eastern Peninsular Ranges to the southeast by a ten lane interstate highway (I-15). We analyzed DNA samples from 97 pumas sampled between 2001 and 2012. Genotypic data for forty-six microsatellite loci revealed that pumas sampled in the Santa Ana Mountains (n = 42) displayed lower genetic diversity than pumas from nearly every other region in California tested (n = 257), including those living in the Peninsular Ranges immediately to the east across I-15 (n = 55). Santa Ana Mountains pumas had high average pairwise relatedness, high individual internal relatedness, a low estimated effective population size, and strong evidence of a bottleneck and isolation from other populations in California. These and ecological findings provide clear evidence that Santa Ana Mountains pumas have been experiencing genetic impacts related to barriers to gene flow, and are a warning signal to wildlife managers and land use planners that mitigation efforts will be needed to stem further genetic and demographic decay in the Santa Ana Mountains puma population.

## Introduction

Genetic diversity, demography, and abundance – biological characteristics that influence population viability – can vary across a species’ distribution. Species that are generally perceived as wide-ranging and abundant are sometimes relegated to status as “least conservation concern”, in spite of indicators signaling concern and frequently, lack of data. Pumas (*Puma concolor*; also known as mountain lion, cougar, and in Florida, panther) epitomize this dilemma. Although pumas in California have not been subjected to hunting since 1972, and were designated as a Specially Protected Mammal in 1990 [1], there is minimal active management and little scientifically validated data on statewide or regional population numbers. Pumas in southern California have one of the lowest annual survival rates among any population in North America, on par with rates seen in hunted populations (unpublished data). They are under increasing threats from habitat loss and fragmentation, and mortality from vehicle strikes, depredation permits, poaching, public safety kills, wildfire, and poisoning [2], [3]. Timely evaluation of potential threats to population viability is imperative in order to prioritize conservation activities to prevent collapse of some populations.

**Figure 1:**
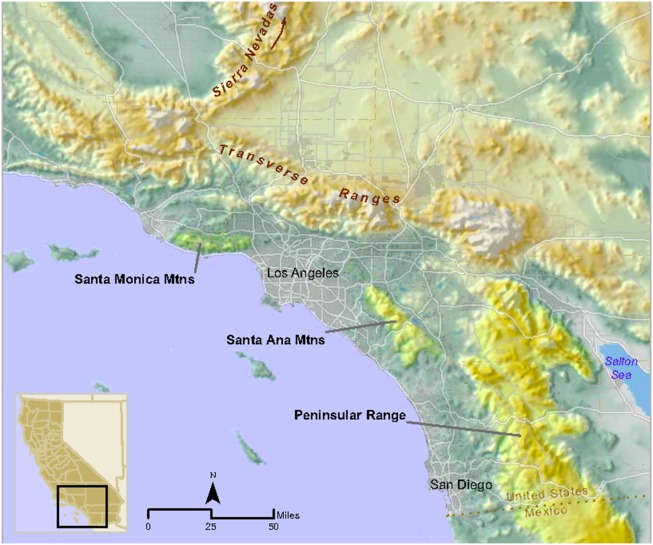
Topographic map depicting location of Santa Ana Mountains, eastern Peninsular Ranges in southern California, and adjacent regions. Inset shows location in the state of California.

The human population of southern California is over 20 million [4] and expected to exceed 30 million by 2060 [5]. This increasing population will likely result in further loss, fragmentation, and degradation of natural habitats in the region. Habitat fragmentation south of greater Los Angeles has effectively turned the Santa Ana Mountain range in mostly Orange and Riverside counties into a ‘mega-fragment’ of habitat, surrounded to the west, north, and east by dense urban land uses. The only remaining montane and foothill habitat linkage connecting the Santa Ana Mountain range to other mountains of the Peninsular Range is a southeasterly swath of habitat bisected by a very heavily traveled 10-lane highway, Interstate 15 (I-15) (Figure 1).

Population viability of pumas in the Santa Ana Mountains (a geography henceforth referred to as distinct from the broader Peninsular Ranges to the east) has been of conservation concern for decades. Population monitoring and modeling in the 1980s highlighted that urbanization and highways were fragmenting puma habitat (e.g., [6]), and that in turn motivated efforts to protect habitat connectivity in the region (e.g., [7], [8]). As part of a statewide assessment of puma genetic diversity and population structure, Ernest et al. [9] employed an 11-locus microsatellite panel and found that, for a limited sample size (n = 14) Santa Ana pumas had lower genetic diversity than other populations in California. Since 2001, pumas in the region have been the subject of an ongoing study by the Karen C. Drayer Wildlife Health Center of the University of California, Davis (UCD) School of Veterinary Medicine. Telemetry data from 74 pumas in the UCD study has confirmed that minimal connectivity (only one GPS-collared puma over ten years was documented to transit successfully; unpublished data) exists between the Santa Ana Mountains and the eastern Peninsular Ranges across I-15, confirming that previous connectivity concerns were warranted.

We conducted a detailed appraisal of the genetic diversity, relatedness, and population structure of southern California puma populations. Using 97 samples collected over 12 years as part of the UCD study, and a 46-locus microsatellite panel, we evaluated levels of genetic diversity, estimated effective population sizes and tested whether genetic data supported a hypothesis of recent bottleneck in the populations. We assessed whether genetics reflected our telemetry observations of infrequent puma crossings of I-15 between the Santa Ana Mountains and the Peninsular Ranges to the east. Additionally we explored inter-population gene flow at multiple time scales by employing methods that reflect recent (a few generations) and more historical (tens or more generations). Finally, we tested our hypothesis that the Santa Ana population had lower genetic diversity than those sampled from other regions in California.

## Materials and Methods

### Samples

**Figure 2:**
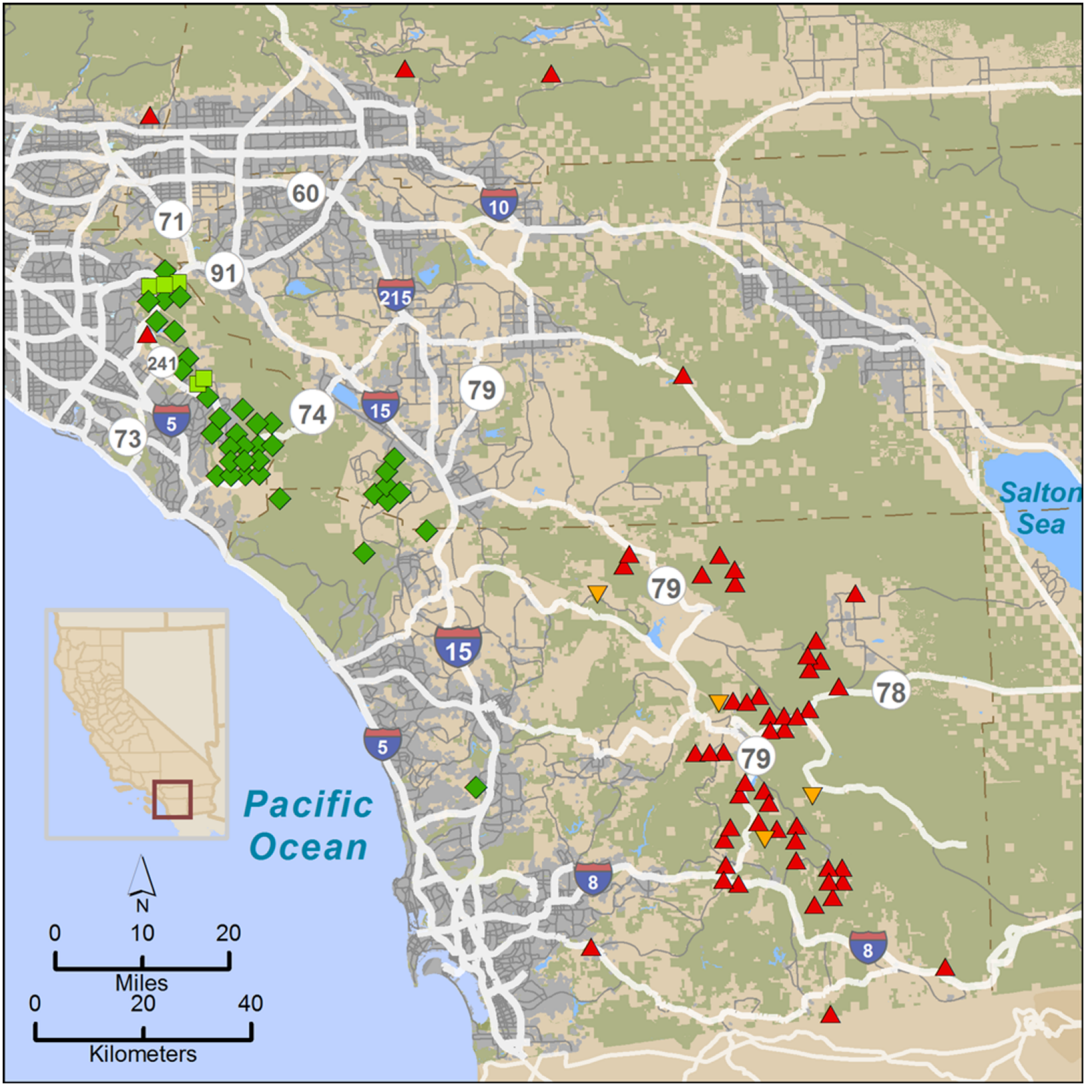
Map of puma capture locations in the Santa Ana Mountains and eastern Peninsular Ranges of southern California. Colors of symbols represent genetic group assignment inferred from Bayesian clustering analysis (STRUCTURE analysis, see Figure 4). Genetic group A-1 = green diamonds; A-2 = red triangles (apex at top). One male puma (M86) captured in the Santa Ana Mountains had predominant genetic assignment to the A-2 (red) genetic group. Five individuals (light green squares) captured in the Santa Ana Mountains had partial assignment to the A-2 group (M91, F92, M93, M97 and F102). Molecular kinship analysis showed that M86 and a female (F89) captured in the Santa Ana Mountains were parents of pumas M91, F92, and M93 (captured in the Santa Ana Mountains). Puma M97 assigned in parentage to M86 and F61, while F102 had unknown parentage (no parentage assignments; due possibly to her death early in project prior to collection of most of the samples). Three individuals (orange triangles, apex at bottom), had partial assignment (however, less than 20%) to A-1.

We obtained blood or tissue samples for analysis of nuclear DNA from pumas captured for telemetry studies, and from those found dead or killed by state authorities for livestock depredation or public safety in San Diego, Orange, Riverside, and San Bernardino counties of southern California (n = 97) during 2001–2012 (Figure 2). Pumas captured for telemetry were captured and sampled as detailed in [10]. Forty-two samples were collected to the west of I-15 in the Santa Ana Mountains, and 55 samples were collected in the Peninsular Ranges to the east of I-15. A small number of additional samples were collected from deceased animals in San Bernardino County just to the north of the Peninsular Range across Interstate Highway 10. For population genetic comparisons with pumas sampled elsewhere throughout California, a 257 sample subset of our statewide puma DNA data archive was employed (regions and sample sizes detailed in Table 1 and depicted in Figure 1 in [9])

**Table 1:** Genetic diversity summary statistics for southern California pumas (n = 97) relative to other populations in California (n = 257).

Sampling Region	Abbrev.		N	Na	AR	Ho	He	I	%P
**North Coast**	**NC**	**Mean**	29	3.6	2.0	0.41	0.44	0.80	98%
		**SE**		0.2	0.1	0.03	0.03	0.05	
**Modoc Plateau & Eastern** **Sierra Nevada**	**MP-ESN**	**Mean**	51	4.2	2.4	0.52	0.54	0.98	100%
		**SE**		0.3	0.1	0.03	0.03	0.05	
**Western Sierra Nevada**	**WSN**	**Mean**	47	4.2	2.4	0.47	0.51	0.95	98%
		**SE**		0.2	0.2	0.03	0.03	0.06	
**Central Coast: north**	**CC-N**	**Mean**	83	3.2	1.9	0.41	0.41	0.70	98%
		**SE**		0.2	0.1	0.03	0.03	0.06	
**Central Coast: central**	**CC-C**	**Mean**	21	3.4	2.1	0.43	0.46	0.81	96%
		**SE**		0.2	0.1	0.03	0.03	0.05	
**Santa Monica Mountains**	**CC-S**	**Mean**		2.2	1.7	0.38	0.33	0.53	76%
**Central Coast: South**		**SE**	26	0.1	0.1	0.04	0.03	0.05	
**Peninsular Range-East**	**PR-E**	**Mean**	55	3.1	2.0	0.43	0.41	0.74	87%
		**SE**		0.2	0.2	0.04	0.04	0.07	
**Santa Ana Mountains**	**SAM**	**Mean**	42	2.3	1.6	0.33	0.32	0.54	80%
		**SE**		0.2	0.1	0.03	0.03	0.05	

Abbrev. = region abbreviations used in Tables and Figures. Mean with standard error (SE). N = sample size. Na = average number of different alleles per locus. AR = allelic richness, standardized to sample size. Ho = observed heterozygosity. He = expected heterozygosity. I = Shannon’s information index (Sherwin et al 2006). %P = percent of polymorphic loci. Regions are detailed further in text and generally follow California Bioregions designations. (http://biodiversity.ca.gov/bioregions.html).

### Ethics Statement

Animal handling was carried out in strict accordance with the recommendations and approved Protocol 10950/PHS, Animal Welfare Assurance number A3433-01, with capture and sampling procedures approved by the Animal Care and Use Committee at the University of California, Davis (Protocol #17233), and Memoranda of Understanding and Scientific Collecting Permits from the California Department of Fish and Wildlife (CDFW). Permits and permissions for access to conserved lands at puma capture and sampling sites were obtained from CDFW, California Department of Parks and Recreation, The Nature Conservancy, United States (US) Fish and Wildlife Service, US Forest Service, US Bureau of Land Management, US Navy/Marine Corps, Orange County Parks Department, San Diego County Parks Department, San Diego State University, Vista Irrigation District, Rancho Mission Viejo/San Juan Company, Sweetwater Authority, California Department of Transportation (CalTrans), and the City of San Diego Water Department.

### DNA Extraction and Microsatellite DNA data collection

Whole genomic DNA was extracted using the DNeasy Blood & Tissue Kit (QIAGEN, Valencia, CA, USA). Fifty microsatellite DNA primers were initially screened for this project. Forty-six loci that performed well in multiplex PCR (using the QIAGEN Multiplex PCR kit; QIAGEN) and conformed to expectations for Hardy-Weinberg and linkage equilibria were selected for ultimate analysis [11], [12], [13]. One sex-identification locus (Amelogenin) was used to confirm sex in samples from degraded puma carcasses [14].

PCR products were separated with an ABI PRISM 3730 DNA Analyzer (Applied Biosystems Inc., Foster City, CA, USA) with each capillary containing 1 µL of a 1∶10 dilution of PCR product and deionized water, 0.05 µL GeneScan-500 LIZ Size Standard and 9.95 µL of HiDi formamide (both products Applied Biosystems Inc.) that was denatured at 95°C for 3 min. Products were visualized with STRand version 2.3.69 [15]. Negative controls (all reagents except DNA) and positive controls (well-characterized puma DNA) were included with each PCR run. Samples were run in PCR at each locus at least twice to assure accuracy of genotype reads and minimize risk of non-amplifying alleles. For >90% samples, loci that were heterozygous were run at least twice and homozygous loci were run at least three times.

### Genetic diversity

The number of alleles (Na), allelic richness (AR; incorporates correction for sample size), observed heterozygosity (Ho), expected heterozygosity (He), Shannon’s information index [16], and tests for deviations from Hardy-Weinberg equilibrium were calculated using software GenAlEx version 6.5 [17], [18]. Shannon’s information index provides an alternative method of quantifying genetic diversity and incorporates allele numbers and frequencies. Testing for deviations from expectations of linkage equilibrium was conducted using Genepop 4.2.1 [19], and we tested for the presence of null alleles using the program ML RELATE [20]. We assessed significance for calculations at alpha = 0.05 and used sequential Bonferroni corrections for multiple tests [21] in tests for Hardy-Weinberg and linkage equilibria.

The average probability of identity (PID) was calculated two ways using GenAlEx: 1) assuming random mating (PID_RM_) without close relatives in a population [22], and 2) assuming that siblings with similar genotypes occur in a population (PID_SIBS_) [23]. Probability of identity is the likelihood that two individuals will have the same genetic profile (genotype) for the DNA markers used. PID_SIBS_ is considered conservative since it probably conveys a higher likelihood; however, we recognized that siblings occurred in these populations.

### Assessing population structure and genetic isolation

We used a Bayesian genetic clustering algorithm (STRUCTURE version 2.3.4 [24], [25]) to determine the likely number of population groups (K; genetic clusters) and to probabilistically group individuals without using the known geographic location of sample collection. We used the population admixture model with a flat prior and assumed that allele frequencies were correlated among populations, and ran 50,000 Markov chain Monte Carlo repetitions following a burnin period of 10,000 repetitions. First, an analysis including 354 statewide puma genotypes (97 from southern California and 257 from other regions) was run to estimate the probability of one through 10 genetic clusters (K), with each run iterated three times. Second, given the output of the statewide run, we ran an analysis using only the 97 southern California puma genotypes to estimate the probability of one through five K, with each run iterated three times. Employing STRUCTURE HARVESTER [26] we averaged log probability of the data given K, log Pr(X|K), statistics across the multiple runs for each of the K estimates. In each case (statewide and southern California), we selected the K value of highest probability by identifying the set of values where the log Pr(X|K) value was maximized and subsequently selected the minimum value for K that did not sacrifice explanatory ability [27], [28], [29]. We defined membership to a cluster based upon the highest proportion of ancestry to each inferred cluster.

To further assess and visualize genetic relationships among regions and individuals, we performed principal coordinates analyses (PCoA) via covariance matrices with data standardization [30] using GenAlEx. This is a technique that allowed us to explore and plot the major patterns within the data sets. The PCoA process located major axes of variation within our multidimensional genotype data set. Because each successive axis explains proportionately less of the total genetic variation, the first two axes were used to reveal the major separation among individuals. Employing Genalex software, a pairwise, individual-by-individual genetic distance matrix was generated and then used to create the PCoA.

Wright’s F-statistic, F_ST,_ was calculated to appraise how genetic diversity was partitioned between populations. As implemented in GenAlEx, we used Nei’s [31] formula, with statistical testing options offered through 9999 random permutations and bootstraps.

### Detecting migrants

We used GENECLASS2 version 2.0.h [32] to identify first-generation migrants, i.e. individuals born in a population other than the one in which they were sampled. Genetic clusters identified during STRUCTURE analysis were treated as putative populations. GENECLASS2 provides different likelihood-based test statistics to identify migrant individuals, the efficacy of which depends on whether all potential source populations have been sampled. We first calculated the likelihood of finding a given individual in the population in which it was sampled, *L_h_*, assuming all populations had not been sampled. We then calculated *L_h_*/*L_max_*, the ratio of *L_h_* to the greatest likelihood among the populations [33], which has greater power when all potential source populations have been sampled. The critical value of the test statistic (*L_h_* or *L_h_*/*L_max_*) was determined using the Bayesian approach of Rannala and Mountain [34] in combination with the resampling method of Paetkau et al. [33]; i.e., Monte Carlo simulations carried out on 10,000 individuals with the significance level set to 0.01.

### Testing for bottlenecks and inferring effective population size

We tested for evidence of recent population size reductions in Santa Ana Mountains and eastern Peninsular Range regions with one-tailed Wilcoxon sign-rank tests for heterozygote excess in the program BOTTLENECK version 1.2.02 [35]. The program evaluates whether the reduction of allele numbers occurred at a rate faster than reduction of heterozygosity, a characteristic of populations which have experienced a recent reduction of their effective population size (Ne) [35], [36]. This bottleneck genetic signature is detectable by this test for a finite time, estimated to be less than 4 times Ne generations [37]. These tests were performed using the two-phase (TPM, 70% step-wise mutation model and 30% IAM) model of microsatellite evolution and 10,000 iterations.

We then estimated contemporary Ne for each of the two regions based on gametic disequilibrium with sampling bias correction [38] using LDNE version 1.31 [39]. Ne is formally defined as the size of the ideal population that would experience the same amount of genetic drift as the observed population [40]. These analyses excluded alleles occurring at frequencies ≤0.05, and we used the jackknife method to determine 95% confidence intervals [38].

### Relatedness analyses: pairwise coefficient and internal

Molecular kinship analysis was conducted using a number of software packages. Pairwise relatedness among individuals was evaluated using the algorithm of Lynch and Ritland [41], with reference allele frequencies calculated and relatedness values averaged within each southern California population, as implemented in GenAlEx. Partial molecular kinship reconstruction was conducted using a consensus of outputs from the GenAlEx pairwise relatedness calculator, ML Relate [20], CERVUS version 3.0.3 [42], and Colony version 2.0.3.1 [43], [44]. Individual genetic diversity (also called internal relatedness) was assessed using Rhh [45] as implemented in R statistical software [46]. This is a measure of genetic diversity within each individual (an estimate of parental relatedness [47], and we averaged over individuals for each of the two regions of southern California. Significance of differences between means was evaluated using t tests.

## Results

Forty-two of the 46 loci that we employed were polymorphic in southern California and selected for the subsequent analyses. The average probabilities of identity with assumptions of either random mating (PID_RM_) or mating among sibs (PID_SIBS_) across the 42 loci for the eastern Peninsular Ranges were (PID_RM_) 6.3×10^−22^ and (PID_SIBS_) 3.1×10^−10^, and for the Santa Ana Mountains were (PID_RM_) 2.8×10^−15^ and (PID_SIBS_) 1.1×10^−7^ respectively. These very small values indicate that the panel of genetic markers provided very high resolution to distinguish individuals. For example, given this data the probability of seeing the same multi-locus genotype in more than one puma was less than one in nine million for Santa Ana Mountains pumas.

### Genetic diversity

Measures of genetic variation including allelic diversity, heterozygosity, Shannon’s information index, and polymorphism, were lower for Santa Ana pumas than most of those tested from other regions of California (Table 1). Such low genetic diversity indicators were approached only by pumas in the Santa Monica Mountains (Ventura and Los Angeles Counties), a neighboring remnant puma population in the north Los Angeles basin (Figure 1).

### Population Structure

**Figure 3:**
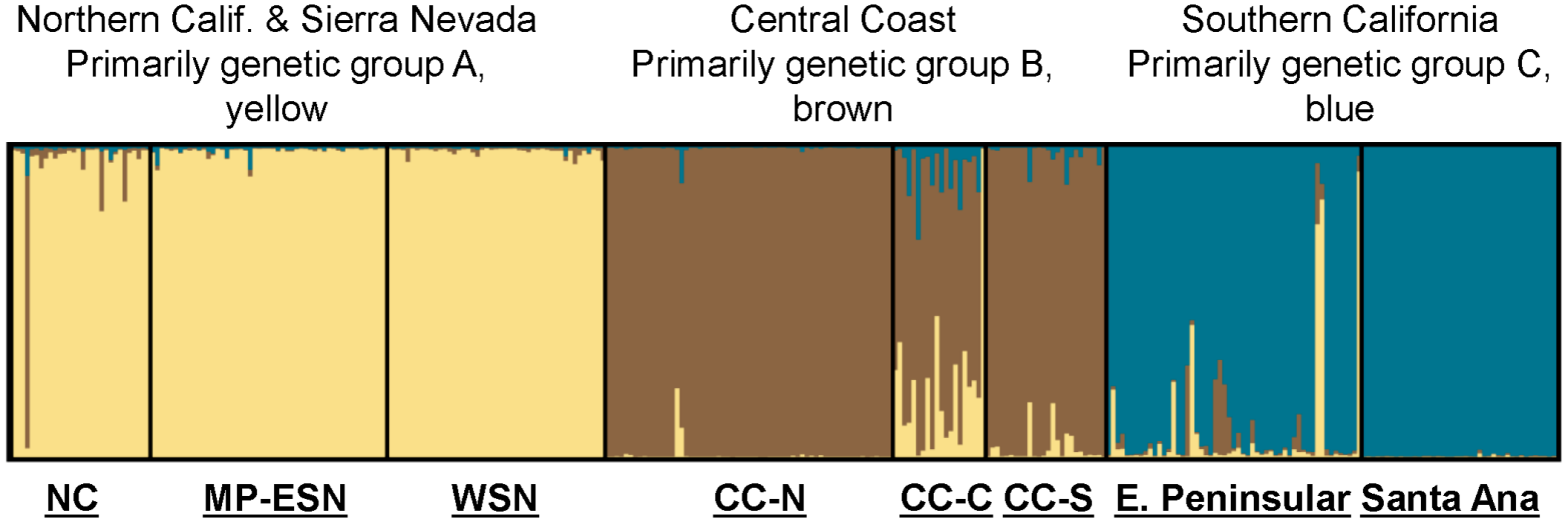
California puma population genetic structure. STRUCTURE bar plot displaying the genetic clustering relationship of southern California pumas relative to others in California. Three major genetic groups, A (blue, on right), B (brown, in center), and C (yellow, on left), are evident for analysis of 354 individuals sampled throughout California. Abbreviations: NC = North Coast, MP-ESN = Modoc Plateau & Eastern Sierra Nevada, WSN = Western Sierra Nevada, CC-N = Central Coast: north, CC-C = Central Coast: central, CC-S = Central Coast: South (Santa Monica Mountains), PR-E = Peninsular Range-East, SAM = Santa Ana Mountains. The plot is organized by grouping individuals in order of their geographic region sampling source. Proportional genetic assignment for each puma is represented by a vertical bar, most easily visualized for pumas that genetically assigned to a group different from most others sampled in its region (for example one individual with over 80% brown and 8% blue near far left of group A). Pumas primarily from the Sierra Nevada Range and northern California are represented by group A (yellow), group B (brown) includes primarily Central Coast pumas and group C (blue) represents primarily southern California pumas (Santa Ana Mountains and eastern Peninsular Ranges).

Bayesian clustering analysis (STRUCTURE; Figure 3 of statewide puma genetic profiles (n = 354), including 97 from southern California, also support genetic distinctiveness of Santa Ana Mountains and eastern Peninsular Range pumas from other populations in the state. Three main genetic groups (A, B, and C) were evident in the analysis (Figure 3) The 97 pumas sampled in southern California (right-hand set of bars in Figure 3, with samples from Santa Ana and eastern Peninsular Range pumas labeled) predominantly cluster within genetic group C. The Santa Ana pumas assign very tightly to group C (0.996 average probability assignment), while pumas of the eastern Peninsular Ranges showed more variable assignment (0.93 average probability assignment), with 9 individuals (16%) having less than 0.90 assignment. Pumas sampled in the Central Coast of California (which included Santa Monica Mountains pumas) make up the central set of bands, and those individuals predominantly assign to the genetic group B. Pumas sampled in the other regions of California (North Coast Ranges, Modoc Plateau, western Sierra Nevada, and eastern Sierra Nevada) predominantly cluster with the genetic group A. Notably, there are individuals sampled in each geographic area which cluster with a genetic group that is not the dominant one in that area, suggesting dispersal events and/or genetic exchange that have occurred to varying degrees in each region.

**Figure 4:**
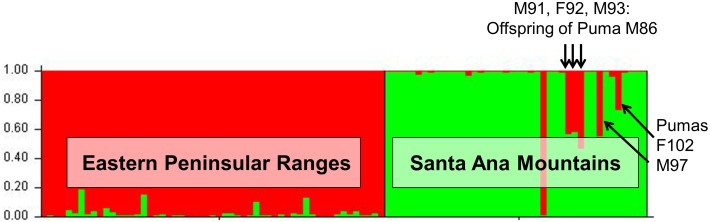
Southern California puma population genetic structure. Bar Plot displaying results of STRUCTURE analysis focused on genotypic data from 97 southern California pumas (the blue block from Figure 3). With removal of the strong genetic signal from northern California and Central Coast samples (see Figure 3), two distinct southern California groupings were inferred, C-1 (green, on right) and C-2 (red, on left). These reflect the two regions: Santa Ana Mountains to the west of I-15 (predominantly genetic group C-1) and eastern Peninsular Ranges to the east of I-15 (predominantly genetic group C-2). Genetic clustering is dependent on genetic variance among samples included in the analysis. One male puma (M86) captured in the Santa Ana Mountains has predominant genetic assignment to the C-2 (red) genetic group (the predominant genetic cluster for PR-E), and five others had partial assignment to the C-2 group (M91, F92, M93, M97 and F102). Molecular kinship analysis showed that M86 and a female (F89) assigning to the C-1 genetic group were parents of pumas M91, F92, and M93 (all were captured in the Santa Ana Mountains).

A STRUCTURE analysis focused only on genetic data from the 97 southern California pumas indicated two distinct genetic groups (C-1 and C-2 shown in Figure 4). Pumas sampled in the eastern Peninsular Range region east of I-15 group primarily with C-2 and those of the Santa Ana Mountain region on the west side of I-15 group with C-1. An exception to the consistent genetic clustering was an adult male (M) puma (M86), that was captured in the Santa Ana Mountains but clustered with pumas from the eastern Peninsular Ranges (primarily genetic group C-2). Five other pumas captured in the Santa Ana Mountains had a 30–50% assignment to the C-2 group (M91, F92, M93, M97 and F102). Molecular kinship analysis showed that M86 and a female (F89) captured in the Santa Ana Mountains and assigned to the C-1 genetic group were the likely parents of three of these pumas (M91, F92, and M93) (results of relatedness and kinship analyses). M86 also was the likely parent of another puma in the group (M97), an offspring of another female (F61) that was sampled in Santa Ana Mountains and clustered with the C-1 genetic group. F102 was a <1 year old female killed by a vehicle in 2003 prior to collection of the majority of samples from adults in the Santa Ana Mountains.

**Figure 5:**
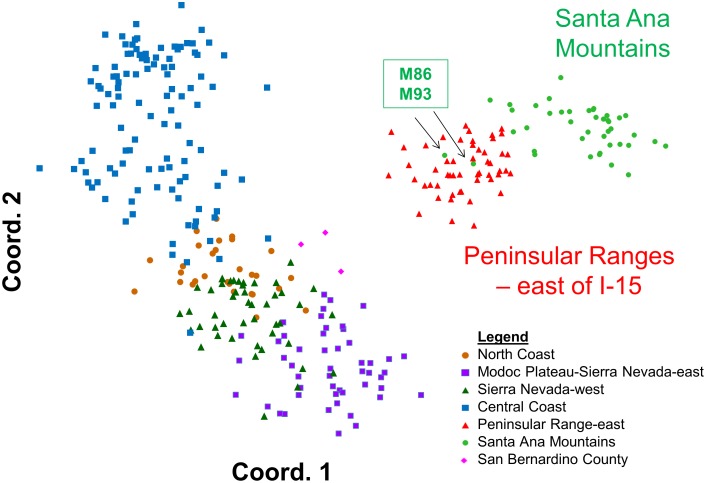
Principal Coordinates analyses (PCoA) constructed using genetic covariance matrices (GenAlEx) for 354 California puma genetic profiles including 97 from southern California. Patterns displayed for first two axes of variation within the genetic data set. Each point, color-coded to its sampling region, represents an individual puma. Note that colors in PCoA diagrams reflect geographic source of samples and not STRUCTURE genetic cluster assignment. Abbreviations and sample sizes per Table 1. Arrows denote pumas described in Figure 4.

**Figure 6:**
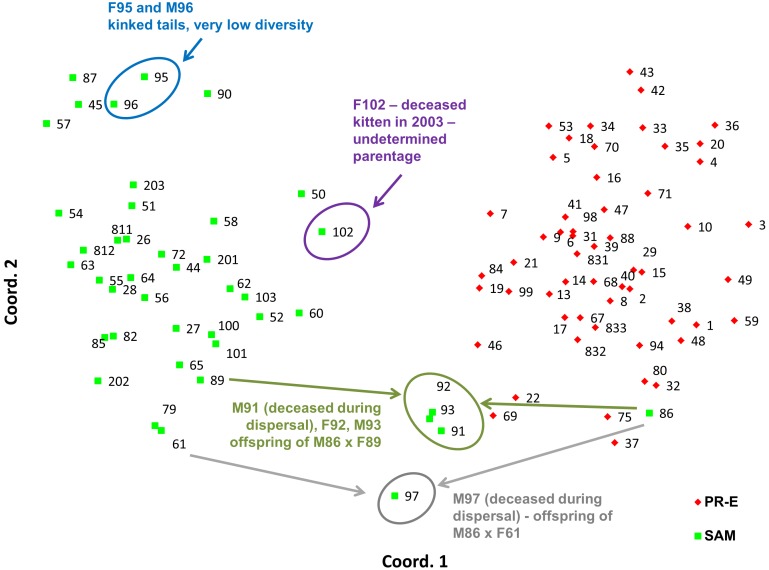
Principal Coordinates analyses (PCoA) via covariance matrices for 97 southern California puma genetic profiles as conducted in GenAlEx. Patterns displayed for first two axes of variation within the genetic data set. Each point represents an individual puma, and has sample identification number and color-coding to sampling region. Note that colors in PCoA diagrams reflect geographic source of samples and not STRUCTURE genetic cluster assignment. Abbreviations and sample sizes per Table 1.

Principal coordinates analysis of statewide puma genetic profiles (n = 354) (PCoA; Figure 5) allowed graphical examination of the first two major axes of multivariate genetic variation, and confirmed and added detail to the genetic distinctiveness of southern California pumas relative to others in California. The PCoA also reinforced the distinctiveness of pumas sampled in the Santa Ana Mountains from those sampled in the eastern Peninsular Ranges. Most pumas sampled in the Santa Ana Mountains align in a cloud of data points distinct from the eastern Peninsular Range pumas, and were the most genetically distant from all other pumas tested in California (Figure 5). The analysis also confirms the STRUCTURE findings that M86 who was sampled in the Santa Ana Mountains genetically aligns with the pumas sampled in the Peninsular Ranges, as does one of his offspring, M93 (see Figure 6 for additional detail). The PCoA position of data points for three pumas sampled in the San Bernardino Mountains north of Peninsular Ranges (pink diamonds in Figure 5) illustrates an intermediate genetic relationship between pumas from the rest of California and pumas sampled in the eastern Peninsular Ranges and Santa Ana Mountains, and suggests that they may represent transitional gene flow signature between southern California and regions to the north and east.

PCoA analysis of only the samples collected in the Santa Ana and Peninsular Ranges (Figure 6) confirms the findings from the STRUCTURE analysis indicating genetic distinctiveness of these two populations despite geographic proximity. Siblings M91, F92, and M93 (offspring of F89 and M86 according to our kinship reconstructions) as well as M97 (likely offspring of a female puma captured in the Santa Ana Mountains, F61, and M86, according to kinship reconstructions) are located graphically midway between their parents’ PCoA locations.

### Genetic isolation

Wright’s F_ST_ calculations (Table 2) indicate that Santa Ana Mountains pumas are the most isolated of those tested throughout California (p = 0.0001). Despite the short distance (as short as the distance across the I-15 Freeway) between the Santa Ana Mountains and the eastern Peninsular Range region, F_ST_ was surprisingly high (0.07) given the very close proximity of the two regions (separated only by an interstate highway). The Santa Monica Mountains pumas and Santa Ana Mountains pumas had the highest F_ST_ (0.27; lowest gene flow) of all pairwise comparisons in the state, demonstrating a high level of genetic isolation between these regions.The Santa Monica Mountains and Santa Ana Mountains are less than 100 km direct distance apart, through the center of Los Angeles. However the more likely distance for puma travel between these two mountain ranges, avoiding urban areas and maximizing upland habitat, would likely exceed 300 km (estimated using coarse measurements on Google Earth, Google, Inc.).

**Table 2:** Wright’s F_ST_ values indicate that southern California mountain lion populations are genetically distinct from other populations in California.

	NC	MP-ESN	WSN	CC-N	CC-C	CC-S	PR-E	SAM
**North Coast (NC)**	0							
**Modoc Plateau & Eastern Sierra** **Nevada (MP-ESN)**	0.09	0						
**Western Sierra Nevada (WSN)**	0.05	0.03	0					
**Central Coast: north (CC-N)**	0.13	0.14	0.12	0				
**Central Coast: central (CC-C)**	0.08	0.09	0.06	0.07	0			
**Santa Monica Mountains (CC-S)**	0.17	0.15	0.12	0.14	0.10	0		
**Peninsular Range-East (PR-E)**	0.13	0.08	0.09	0.12	0.09	0.16	0	
**Santa Ana Mountains (SAM)**	0.18	0.16	0.17	0.19	0.17	0.27	0.07	0

Note that one of the geographically closest puma populations, Santa Monica Mountains, has highest F_ST_ with the Santa Ana population, evidence of high genetic isolation for both regions. Probability, P(random> = data) based on 9999 permutations for all values are <0.001. Abbreviation definitions and sample sizes are included in Table 1.

### Detection of migrants

GENECLASS2 identified four individuals as first-generation migrants (*P*<0.01), four with the *L_h_* method (pumas F75, M80, M86, and M99), and one with the *L_h_*/*L_max_* ratio (M86, which was detected using both likelihood methods). Pumas F75, M80, and M99 were all captured from the San Bernardino Mountains (Figure 2) at the northern extent of the study region, yet clustered with individuals from the Eastern Peninsular Range during STRUCTURE analysis. Their migrant designation may suggest immigration from populations north of Los Angeles and/or a distinct genetic population within the San Bernardino region. Puma M86 was captured in the Santa Ana Mountains, but assigned strongly to the eastern Peninsular Range genetic cluster, indicating a seemingly clear population of origin. This individual assignment is in accord with the clustering results from STRUCTURE (Figure 4).

### Evidence of genetic bottlenecks

The Santa Ana Mountains population exhibited clear evidence of a population bottleneck (Table 3; Wilcoxon sign-rank test for heterozygote excess, and detection of a shift in the allele frequency distribution mode [36]; BOTTLENECK software). The eastern Peninsular Range mountain lions did not show a strong signature of a bottleneck.

**Table 3:** Effective population size estimations and indications of recent genetic bottlenecks in southern California pumas.

	Mode	TPM	Ne (P-CI; JK-CI)
Santa Ana Mtns	Shifted mode	0.009	5.1 (3.3–6.7; 3.3–6.6)
Peninsular Range, East	Normal L	0.19	24.3 (21.7–27.3; 20.6–28.8)

Listed by column are p-values for population bottleneck tests (Wilcoxon sign-rank test; BOTTLENECK) assuming the two-phase (TPM) model of microsatellite evolution. Effective size (Ne) estimations (95% CI) based on data from 42 microsatellite loci. The Santa Ana Mountains population exhibited clear evidence of a population bottleneck. Effective population size estimate using the point estimate linkage disequilibrium method of (LDNE, Waples 2006) with 95% confidence intervals (CI) for both parametric (P) and jackknifed (JK) estimates.

### Effective population size

Effective population size (Ne) estimations using the linkage disequilibrium method (LDNe program) were 5.1 for the Santa Ana Mountains population and 24.3 for mountain lions in the eastern Peninsular Ranges. Statistical confidence intervals for both regions, given the genetic data, were tight (Table 3).

### Relatedness: pairwise coefficient and internal

**Figure 7:**
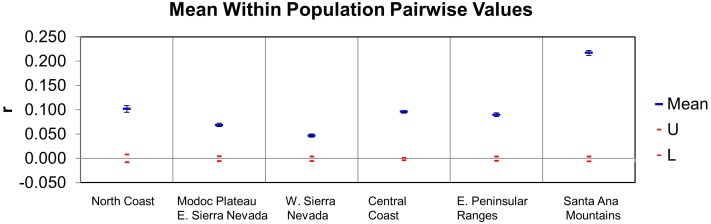
Average pairwise relatedness (r; blue bars with confidence intervals) for pumas sampled in southern California relative to other regions in California. Algorithm of Lynch and Ritland (1999) as implemented in GenAlEx. Expected range for “unrelated” is shown as red bars with confidence intervals. The average relatedness of Santa Ana Mountain pumas is higher than those sampled in Peninsular Ranges east of I-15 and for any other region tested in California. Relatedness in the Santa Ana Mountains pumas approaches second order family relationship (half sibs, niece-aunt, grandparent-grandchild, etc.). Abbreviations listed in Table 1.

The average pairwise coefficient of relatedness (r, Figure 7) was highest in Santa Ana Mountains pumas relative to all others tested in California (0.22; 95% confidence interval of 0.22–0.23), a level that approaches second order kinship relatedness (half-sibs, grantparent/grandchild, aunt-niece, etc). The value for the eastern Peninsular Ranges was 0.10 (confidence interval of 0.09–0.10), less than that of third order relatives (first cousins, great-grandparent/great grandchild). Other regions of California averaged similar or lower values to those of eastern Peninsular Ranges (Figure 7).

**Figure 8:**
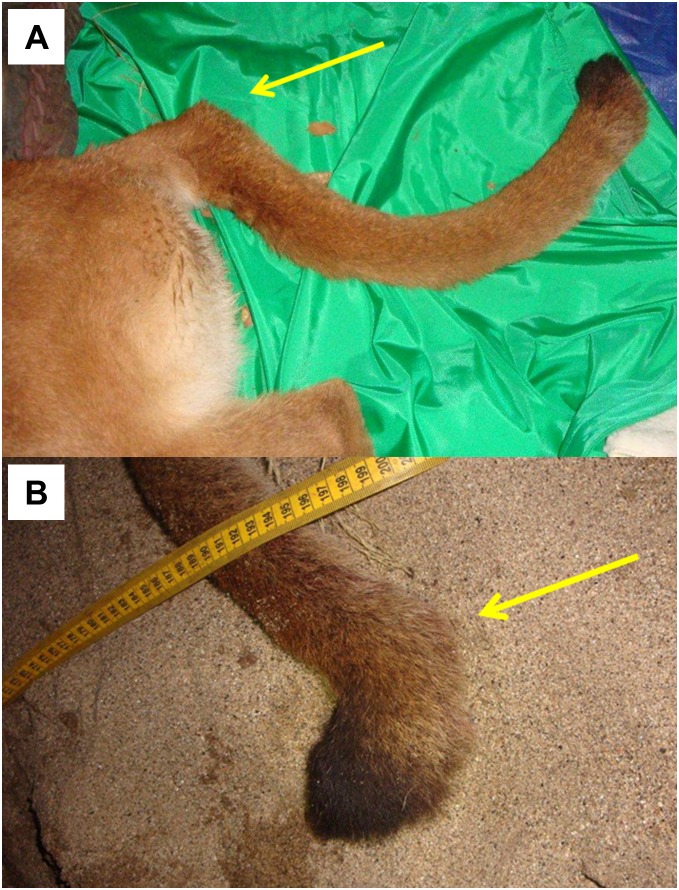
Photographs of kinked tails of pumas F95 (a) and M96 (b). Arrows indicate kink sites. Puma F95 had tail kink at base of tail and Puma M96 had tail kink near distal tip of tail. These two pumas had among the lowest genetic diversity measured in this study.

Among pumas sampled in the Santa Ana Mountains, the population average (0.14) for internal relatedness as implemented in rHH software was significantly higher (t test; p = 5.8×10^−6^) than for those sampled in the eastern Peninsular Ranges (0.001). Of a group of six pumas which clustered near one another in PCoA (Figure 6), five have among the lowest individual genetic diversity measured in southern California (Puma ID [Internal Relatedness value: F45 [0.37], F51 [0.37], M87 [0.28], F90 [0.21], F95 [0.38], and M96 [0.33]). Notably, pumas F95 and M96 (highest internal relatedness) were observed with kinked tails at capture in the Santa Ana Mountains (Figure 8).

## Discussion

Pumas of the Santa Ana Mountains are genetically depauperate, isolated, and display signs of a recent and significant bottleneck. In general, coastal California puma populations have less genetic diversity and less gene flow from other populations than those farther inland [9] (Table 1). This study showed that two coastal populations (Santa Ana Mountains and Santa Monica Mountains) had particularly low genetic variation and gene flow from other regions. Lack of gene flow is likely due in part to natural barriers to puma movement: geography and habitat (Pacific Ocean to the west; less hospitable desert habitat bounding certain regions, etc.). However, our data suggest that anthropogenic developments on the landscape are playing a large role in genetic decay in the Santa Ana Mountains puma population. As large solitary carnivores with sizable habitat requirements, pumas are extremely sensitive to habitat loss and fragmentation [48], [49].

The genetic bottleneck in the Santa Ana Mountains pumas is estimated at less than about 80 years, depending on definitions of effective population size (Ne) and puma generation time. Luikhart and Cornuet [37] state that the bottleneck signatures decay after “4 times Ne [here estimated to be 5.1] generations”. Logan and Sweanor [50] estimated generation time for their New Mexico population of pumas to be 29 months (2.4 years) for females. If an allowance of 2.4–4.0 years is made for generation times (unknown) in the Santa Ana Mountains population, the maximum estimated time since a bottleneck would be about 40–80 years. This was a period of tremendous urban development and multi-lane highway construction in southern California, particularly I-15 [51]. It is likely that the potential for connectivity between the Santa Ana Mountains and the Peninsular Range-East region will continue to be eroded by ongoing increases in traffic volumes on I-15, and conversion of unconserved lands along the I-15 corridor by development and agriculture [8], [48], [52].

An isolated population of pumas in the Santa Monica Mountains to the north of the Santa Ana Mountains also exhibit low values relative to other western North American populations (see Table 2 in [53]. Santa Monica pumas are isolated by urbanization of a megacity and busy wide freeways (Ventura county, including greater Los Angeles region [53]. Multiple instances of intraspecific predation, multiple consanguineous matings (father to daughter, etc.), and lack of successful dispersal highlight a suite of anthropogenic processes also occurring in the Santa Ana Mountains. Our collective findings of kinked tails *and* very low genetic diversity in Santa Ana pumas F95 and M96 may portend manifestations of genetic inbreeding depression similar to those seen in Florida panthers [54], [55]; however recognizing that kinked tails can have non-genetic etiologies.

Our analyses suggest that the Santa Ana Mountains puma population is highly challenged in terms of genetic connectivity and genetic diversity, a result hinted at in Ernest et al. [9] and now confirmed to be an ongoing negative process for this population. This compounds the demographic challenges of low survival rates and scant evidence of physical connectivity to the Peninsular Ranges east of I-15 (unpublished data). Beier [6] documented these same challenges during the 1990’s, and data from the ongoing UCD study suggest the trends have accelerated. Substantial habitat loss and fragmentation has occurred and is continuing to occur; Burdett et al. [10] estimated that by 2030, approximately 17% of puma habitat that was still available in 1970 in southern California will have been lost to development, and fragmentation will have rendered the remainder more hazardous for pumas to utilize. Riley et al [53] document a natural “genetic rescue” event: the 2009 immigration and subsequent breeding success of a single male to the Santa Monica Mountains. This introduction of new genetic material into the population was paramount to raising the critically low level of genetic diversity, as also exemplified by the human-mediated genetic augmentation of Florida Panthers with Texas puma stock [56].

These findings raise concerns about the current status of the Santa Ana Mountains puma population, and the longer-term outlook for pumas across southern California. In particular, they highlight the urgency to maintain – and enhance – what connectivity remains for pumas (and presumably numerous other species) across I-15. Despite warnings [6], [9] about potential serious impacts to the Santa Ana Mountains puma population if concerted conservation action was not taken, habitat connectivity to the Peninsular Ranges has continued to erode. We are hopeful that these new genetic results will motivate greater focus on connectivity conservation in this region. Indeed, the Santa Ana Mountains pumas may well serve as harbingers of potential consequences throughout California and the western United States if more attention is not paid to maintaining connectivity for wildlife as development progresses.
